# Editorial Correspondence of the Charleston Medical Journal and Review

**Published:** 1853-07

**Authors:** 


### SELECTIONS.

*Editorial Correspondence of the Charleston Medical Journal and Review.*

*(Concluded from last Number.)*

I saw M. Guersant, who has charge of the surgical division of the Hospital for sick children, puncture a tumour, in a girl aged six years, situated on the inner and inferior side of the thigh at its pelvic extremity just beneath the sartorious muscle, from which he drew a large quantity of pus. It was elevated, about four inches in diameter, and presented precisely the appearance and feel of a fatty tumour. It had existed for more than a twelvemonth, succeeding caries, ulceration and curvature of the spine, the pus as it formed escaping by gravitation through the femoral ring, or rather under Poupart's ligament. M. G. had removed one of these in the same way from a man in whom it had existed eighteen years. Their contents, it is well known, are enveloped in a membrane which invests the walls of the tumor, in this way preventing its exit by an artificial opening externally, while at the same time no absorption occurs. There was in his wards a case of external Balanitis in a boy, æt. 5, which had extended itself to the urethra by continuity of structure, and which exactly simulated gonorrhœa. The pus (escaping on pressure) was yellow. This condition, which proceeded simply from want of cleanliness, suggests to us how easily one may be deceived in ascribing it to the effect of actual contact. We read in the journals occasionally of very little boys, who either from extraordinary precocity on their part, or owing to the vicious interference of individuals of the other sex, suffer from diseases reserved for their seniors.

Guersant treats white swelling of knee joint (Hydrarthrus synovialis) very successfully, by passing the actual cautery over the surface, and repeating it every two days. He has invited attention recently to Cervical Rheumatism, or arthritis of the neck, a rheumatic condition of the periosteum and the deep-seated recti muscles of this region, and not the sterno-cleidomastoid as is usually supposed. It is successfully treated with the hydriodate of potash internally, and the external application of Vienna caustic. I saw two cases in his wards of individuals of six and seven years of age respectively. One of these, a boy, could not raise his head from the pillow without first supporting and lifting it with one hand. Under the course of treatment indicated above, the power over the voluntary muscles was daily increasing and the pain diminishing. I saw him during one morning visit his wards, continue a clinical lecture on tracheotomy in croup, with the exhibition of the instruments and their mode of application—perform two operations for lithotrity in boys, æt. five and eight—cauterize an erectile tumour of face, with the needle at white heat repeatedly passed into it, in the person of a little girl of nine years of age, and operate on another for tenotomy, dividing the tendons of each foot. Chloroform was administered in each, with the exception of that of erectile tumour. In the latter there was comparatively little pain endured. M. Guersant next exhited the diseased organs of a boy æt. eight, who died of a tumour of the bladder. It had been accompanied with retention of urine for the preceding six months, and intense pain, and susceptibility upon pressure of the abdomen. The organ could not be sounded with the ordinary instrument, but required a much longer one. In and around the interior of the neck of the bladder was found a polypous-shaped encephaloid tumour, which resembled a fibro-plastic structure, not yet determined, and continuing itself along the urethra, involving the prostrate gland. The kidneys, which he had not expected to find diseased, were much altered, and resembled the spleen in its general appearance.

On the general subject of Demonstrative Midwifery (though this is not exactly it, and to give a portion of our readers some idea of the *realities* of medical life in Paris, where an individual is privately watched and publicly permitted to do as he pleases, or *vice versa*,) I will extract a passage from my note-book, as it was written on the spot: "To-day, Jan. 23, at No.—, Rue Poitevin, we are attending M. P.'s *Cours d'Accouchement*, which is open every day, Sundays and Thursdays excepted. It continues from three to four months, and each pays the small sum of twenty-five francs, including the fee of the *toucher*, with version and other obstetrical manipulating operations upon the manikin. It is a small room, filled with students and graduates of medicine, native and foreign. We have now arrived at that portion of the course devoted to the examinations, and M. P. has before us four women in different stages of gestation, and one not *enciente*, which allows of comparison. Each student in turn, touches and explores carefully the latter, under the immediate direction of the chief, and then proceed alone to examine the others. One of these informed one of us, that she received two francs for the use of her person on each occasion, and that she sometimes attended more than one room of a similar nature. M. P. is quite impressive. His subject, a rather respectable looking personage of thirty-two, is standing against the wall looking camly on, and with an occasional smile and exclamation upon the crowd of men around her eager to practice manipulations, a knowledge of, and skill in performing which, are so essential to their art. There are, also, standing or being seated indiscriminately among the rest of us, two or three well-dressed young women, denominated *Sages femmes*, who come to learn the principles and the practice of midwifery, having before them, perhaps, as incentives, the career of their illustrious predecessors, Mesdames Boivin and La Chapelle. These smile like the rest when anything occurs through the awkwardness of some or maladresse of others. Generally, however, there is a good deal of propriety observed, though neither M. P. nor the *Eleves* seem to consider it incumbent upon them to be particularly reserved. He told subject No. 1 (*non-enciente*) not to laugh, *because*, if she did, the Monsieur could not appreciate the differences in the 'neck' as well.

A man of any sensibility, in availing himself, under the circumstances, of these opportunities to render himself more competent and skilful in the practice of certain branches of his profession, and for the benefit ulteriorly of himself and others, cannot, at the same time, but feel pity for the miserable creatures who, reduced by want, or made callous by habit, turn the peculiar condition in which they find themselves, into a means of procuring the necessaries of life, or into a source of gain.

If our readers will pardon me for the nonce, I add: verily, there might be an "Uncle Tom's Cabin" written on this and other conditions existing here, by those who are dealers and retailers in fiction; for the amount of fact really existing, would furnish quite as much foundation for just as much false sentiment, distortion and extravagance, which first captivating, then perverts and leads astray the judgement.

Of course all medical men, as well as students of medicine, who desire it, are admitted to witness and attend the deliveries which daily occur at the Clinique for this purpose at the Ecole de Medecine. The suspension of the green lantern at its front, to indicate the commencement of the throes of parturition in the females who go to seek relief, is discontinued, but only temporarily, and on account, I believe, of the epidemic of puerperal fever.

We should, perhaps, add, respecting the *Sages femmes*, that they undergo a very strict examination before being allowed to have charge of lying-in women; and they sign an obligation, under heavy penalties for its infraction, never to practice what may be denominated an obstetrical operation requiring instruments.

Although the opinion of a physiologist as distinguished as M. Coste are well known to many of the profession, yet as he is at present delivering a course of lectures at the College of France, on *Embryology*, the expression of his very latest views, even at the risk of some repetition, may not be without interest. I will, therefore, condense what I have heard him say on the subject—of which, however, he is at present on at the threshold. Of the two or three introductory lectures on the Artificial Reproduction of Fish in the Waters of France, by the external contact of the ova of the female and the fluid of the male, I send you a more detailed account.

Coste himself, Pouchet, Barry, Wagner and Baer, and we may add Prof. Agassiz, (whom our country is so fortunate as to possess as a resident,) have certainly contributed more than any others, living or dead, to the removal of the barrier existing between us and the laboratory of nature. They have beaten down more of the outworks, and penetrated deeper and farther in the attempt to surprise nature herself in one of the most mysterious and elementary of her operations. Notwithstanding, we must acknowledge that they have only succeeded in gaining the vestibule to her inner shrine, and have not yet exposed the secrets of her mechanism. M. Coste, basing his conclusions upon experiment, believes that in the higher animals, and probably in man, impregnation may take place in the ovary, but always below the second third of the fallopian tube, and not in the matrix (uterine cavity), in this respect differing from some other physiologists. In the procreative seminal fluid of the male, he recognizes the two components, viz: spermatozoa and fluid. The former are the active agents, and are found in a few hours after the congress of the sexes, in the matrix, in the fallopian tube at its fimbriated extremity, and even upon the surface of the albuminous covering of the germinative body. They can only be active in fecundating the germ within the limits indicated above—that is, at any point within the lower third of the fallopian conduit; because experiments upon, and examinations of rabbits, prove that when a germ in a female, from the presence or proximity of the male, or other natural cause, has had its surrounding vesicle ruptured, and has reached this portion without meeting the male animalcule, it has become so disorganized or modified as no longer to possess the power of constituting an individual. Mr. Coste thus demonstrated this: Female rabbits, at the period of rut (which, as well as menstruation in our own species, is nothing more than the evidence of the sympathetic irritation and peculiar state of susceptibility of an animal, induced by the rupture of a matured Graafian vesicle), were placed in the neighbourhood of the male, but not in actual contact; a period of time was allowed to elapse sufficient to bring about a rupture of the vesicle, the seizure of the ovum and its surrounding granular mass by the fimbriæ of the fallopian tube, and its transit into the matrix. If ingress was permitted to the male at any period so late as that a union between the spermatozoa, and the germ or *egg*, as it is more properly called, could not occur before the point referred to above, no conception took place. If the male was denied, to a still later period, the female lost all further desire, and would repulse her mate. Ablation of the ovaries will consequently destroy the capability of procreating, and in our own species of menstruating. Almost the only positive proof, and one well known sustaining the extension of this principle, which is deduced pretty much from comparative experiments on the lower animals, was that obtained by Mr. Percival Pott, who, in extracting an abdominal tumour, took away at the same time with it both ovaries. The subject, who was within the period of puberty, sustained a change quite analogous to that following castration in the opposite sex. She never afterwards menstruated—became, in many respects, masculine in character and disposition, and also underwent corresponding alterations in her muscular system. A cat, at the period of rut, kept in the neighboorhood but free from the encroachment of the male, suffers from marasmus, unless she has been previously deprived of her ovaries. Certain viviparous fish may also be instanced, in which conception, impregnation and gestation are all conducted in the ovaries. So the ovaries are the centre or *foyer* of these emotions, induced primarily by, and attended with, a rupture of a Graafian vesicle. Sexual instincts and their incentives, are the primary cause of this rupture. This rupture gives exit to a structure endowed with unusual attributes, and intended for a wonderful purpose, which is made up of the following vesicle elements, viz: a vitellus and its membrane, a germinative body of Purkingian corpuscle, and a germinative dot.

Conception cannot be, as some physiologists believe, sudden and instantaneous. In man, the opinion is, that it requires ten or eleven days for the fecundation of the germ, and its deposit in the cavity of the uterus, the animalcule having made the traverse of matrix and oviduct to impregnate it. In tracing the progress of the spermatozoa, it may be that they reach their destination by means of the cilia lining the walls of the uterus, the extension of the same combined with a species of suction ill understood, brought about by the action of the muscles of the tube, which assist in conveying them to its distal extremity. When they have reached this point, having found a germ either in the ovaries or already at the vestibule of the tube, they are absorbed into the albumen surrounding it. This is shown by the examination of the higher animals, and is witnessed in the external generation of frogs, and specimens of the various states were exhibited. M. Coste does not agree with physiologists who describe the animalcule as penetrating into the substance of the germ itself, by a cavity or fissure. They have been led into the error by an optical deception, produced by the pressure which the preparation for the microscope gives rise to. It is by a process of imbibition simply into the albuminous covering, that it touches the *surface* of the germ. The egg, thus impregnated, is now borne upwards through the tube and into the matrix, which, in the rabbit, requires twelve hours. When it reaches this point, the changes that it has undergone are simply these: It has lost its envelope of albumen, absorbed for its nourishment, and the animalcule which had been sunk in the latter, is discovered in contact with the vitteline membrane, covering the cicatricula or germinative dot.

The presence of the animalcule is necessary to modify the germ; for, as was stated before, the latter is disorganized if, in its passage outwards, it does not meet the tribute contributed by the male below a certain point in the oviduct. The line which appears at this period on the germ, is only an indication of the commencing *segmentation*, as it is called, for the *yellow body* which is a process of development in itself. It is not, as some physiologists believe, the line of demarcation of the cerebro-spinal system.

M. Coste could find no membrane lining the cavity of the matrix, as some suppose, to keep the impregnated egg which had arrived there from falling out, or to attach it to the walls. He had examined a woman who had committed suicide after impregnation, in whom there existed simply a tumefied membrane unclosed, which was exhibited.

The American Medical Society in Paris, is generally well attended. It receives most of the journals—American and European—and, I think, is productive of much benefit. It is with much pleasure that I inform you of the honor conferred upon our distinguished fellow countrymen, Valentine Mott, M.D., of New York, and J. C. Warren, M.D., of Boston, who have been made Foreign Associates of the Imperial Academy of Medicine. Prof. Simpson of Edinburgh, among four or five others, also shared in this distinction.

Among the candidates mentioned in the Gazette des Hopitaux, for the chair of Materia Medica and Therapeutics, vacated by M. Trousseau, are MM. Grisolle, Tardieu, Pedoux, Fleury and Cazenave. For that of Natural Medical History, MM. Payer, author of the Botanique Cryptogamique, Robin, Hœffer and Martins.

The cholera is at Breslau.

I shall send you an analysis of a pamphlet on Cancer, recently published by M. Maissonneuve; and, in conclusion, I hope to date my next communication on the banks of the "yellow Tiber," or at the Pass of Thermopylæ.

